# Adults Scan Own- and Other-Race Faces Differently

**DOI:** 10.1371/journal.pone.0037688

**Published:** 2012-06-01

**Authors:** Genyue Fu, Chao S. Hu, Qiandong Wang, Paul C. Quinn, Kang Lee

**Affiliations:** 1 Department of Psychology, Zhejiang Normal University, Jinhua, China; 2 Department of Psychology, University of Delaware, Newark, Delaware, United States of America; 3 Dr. Eric Jackman Institute of Child Study, University of Toronto, Toronto, Canada; 4 Department of Psychology, University of California San Diego, La Jolla, California, United States of America; University of Leuven, Belgium

## Abstract

It is well established that individuals show an other-race effect (ORE) in face recognition: they recognize own-race faces better than other-race faces. The present study tested the hypothesis that individuals would also scan own- and other-race faces differently. We asked Chinese participants to remember Chinese and Caucasian faces and we tested their memory of the faces over five testing blocks. The participants' eye movements were recorded with the use of an eye tracker. The data were analyzed with an Area of Interest approach using the key AOIs of a face (eyes, nose, and mouth). Also, we used the iMap toolbox to analyze the raw data of participants' fixation on each pixel of the entire face. Results from both types of analyses strongly supported the hypothesis. When viewing target Chinese or Caucasian faces, Chinese participants spent a significantly greater proportion of fixation time on the eyes of other-race Caucasian faces than the eyes of own-race Chinese faces. In contrast, they spent a significantly greater proportion of fixation time on the nose and mouth of Chinese faces than the nose and mouth of Caucasian faces. This pattern of differential fixation, for own- and other-race eyes and nose in particular, was consistent even as participants became increasingly familiar with the target faces of both races. The results could not be explained by the perceptual salience of the Chinese nose or Caucasian eyes because these features were not differentially salient across the races. Our results are discussed in terms of the facial morphological differences between Chinese and Caucasian faces and the enculturation of mutual gaze norms in East Asian cultures.

## Introduction

How we process the faces of own- and other-races similarly or differently has been one of the enduring topics in psychology and neuroscience [1]–[3]. This question has received extensive empirical investigation since the early 1900s [4], in part because the answers may elucidate a host of important issues in cognitive and social psychology, such as the role of experience in the formation of visual processing expertise and the origin and establishment of racial prejudice and stereotypes [1]–[3], [5], [6].

It is now well established that individuals process faces from different races differently. One of the manifestations of such differential processing is the so-called other-race effect (ORE) of face recognition: Individuals generally recognize own-race faces more accurately and faster than other-race faces. The ORE is highly robust. It has been observed among individuals from different ethnicities and countries, who are not only adults [1]–[3], [7], but children [8], [9] and even infants [10]–[15], [16]. Using event-related-potential and functional MRI methodologies, investigators have also examined the neural mechanisms underlying the ORE in the temporal and spatial domains. For example, researchers have found that the amplitude of the N170, a negative potential in the posterior scalp sites putatively related to face processing (i.e., at the occipito-temporal (P7/8 and PO7/8) sites) is of lower amplitude when viewing upright own-race faces than when viewing other-race faces [17], suggesting that the ORE takes place quickly at about 170 ms post stimulus onset. Golby, Gabrieli, Chiao, and Eberhardt, using fMRI, found that the bilateral middle fusiform areas, which are highly responsive to faces, had greater activations for own-race faces than other-race faces, and the activations within the left fusiform area were positively correlated with the magnitude of the same-race recognition advantage [18]. Using a novel temporal analysis technique, recently, Natu, Raboy, and O'Toole [19] found that the greater responses to own-race faces relative to other-race faces were mainly in the early stage of stimulus presentation.

Whereas the existing behavioral and neural imaging studies have provided insight regarding factors that may contribute to the size of ORE (e.g., age of participants, extent of contact) and its underlying cognitive and neural mechanisms, surprisingly little is known about how individuals visually scan own- and other-race faces. Evidence of different patterns of own- and other-race face viewing may elucidate visual strategies used by observers for encoding and recognizing faces from categories with which one has or does not have visual expertise or that include in- or out-group members. Eye-tracking is one of the ideal methodologies for such purposes because it allows for recording the fixation of various observers (e.g., old or young, normal or impaired) on various parts of the face in real time with relatively high temporal and spatial resolution [20], [21].

There have been several recent studies that have used the eye-tracking methodology to examine how individuals visually scan own- and other-race faces. It has been found that Caucasian and Chinese university students matriculating at the same Scottish university scanned photographs of own- and other-race faces differently [22]. While all participants scanned the major internal facial features regularly (i.e., the eyes, nose, and mouth), Caucasian students tended to focus on the eye regions, whereas Chinese students tended to focus on the nose. Caldara and his colleagues [22] have argued that the difference in visual scanning pattern may be due to the fact that Western observers prefer analytic perceptual strategies, whereas East Asians prefer holistic perceptual strategies. Western Caucasians focus more on the eye region which provides perhaps the most crucial featural information about the face, whereas East Asians focus more on the nose region which is the optimal point in the face for obtaining and integrating all information on the face holistically.

Somewhat parallel to these adult findings, recent evidence reported by Lee, Quinn, and their colleagues [23], [24] suggests that the differential scanning strategies by Chinese and Caucasian observers may emerge as early as infancy. Wheeler et al. [24] recorded the visual fixations of Caucasian infants between 6 and 10 months of age when viewing a dynamic display of an own-race Caucasian face vs. that of an other-race African face. They found that with increased age, infants' visual attention to the eye regions of the own-race faces increased significantly, whereas their visual attention to the other-race eyes did not change. In contrast, Liu et al. [23] found that when viewing own-race Chinese and other-race Caucasian faces, Chinese infants' fixation time on the Chinese nose has no significant change [24], whereas their fixation time on the Caucasian nose decreased significantly with increased age. It seems that Caucasian and Chinese infants have differential scanning patterns for own- and other-race faces. This difference has been suggested to stem from cross-cultural differences in face-to-face interaction between parents and infants: Western parents are significantly more likely to call their infants to make eye contact than Chinese parents [25].

It should be noted, however, that there exists a marked difference between the findings of the recent infant studies and the findings of Caldara and his colleagues [22]: Caldara and his colleagues found that although Caucasian and Chinese adults use different scanning strategies (more focus on the eyes by Caucasians and on the nose by Chinese), they do so for both own- and other-race faces. In contrast, with increased age, the Caucasian infants in Wheeler et al. [24] increasingly focused on the eyes of the own-race Caucasian faces, whereas the Chinese infants in Liu et al. [23] increasingly focused on the nose of the own-race Chinese faces. Aside from the age differences between the participants in these two sets of studies and the use of dynamic face images for infants vs. static face images for adults, one possibility is the extent of other-race experiences among the participants in the studies by Lee and his colleagues and those in the studies by Caldara and his colleagues [22]. In the former studies, the participants had not had direct contact with other-race faces, whereas in the latter studies, the Chinese participants (as well as the Caucasian participants) were university students attending a Scottish university with many international students. Thus, the other-race experience by participants in the study by Caldara and his colleagues, albeit limited, might be sufficient to allow them to generalize their culture-specific scanning strategy developed for own-race faces to scan other-race faces. If this possibility is true, one should expect individuals with no direct contact with other-race individuals to use their culture-specific scanning strategy to scan only own-race faces. The present study specifically tested this possibility.

To test this hypothesis, we recruited Chinese adults who had never had direct contact with other-race individuals. We first showed them a set of Chinese and Western Caucasian faces to be remembered. After initial familiarization, we showed these target faces again along with an equal number of new Chinese and Caucasian foil faces, one at a time. The participants were asked to indicate whether the face seen was a familiarized face (target face) or a foil face (foil face). After this recognition test, participants were immediately told whether their response was correct or incorrect. Regardless of participants' accuracy, we showed the target face again for participants to review. This test-feedback-review cycle was repeated until all target faces and foil faces were shown, after which the test-feedback-review cycle was repeated for an additional 4 blocks. The 5 test-feedback-review blocks served to examine whether participants' responses and eye movement patterns would change as they became more and more familiarized with the target faces. We used an eye tracker to record the participants' fixations on the faces during familiarization and the tests and reviews. We hypothesized that Chinese adults without any direct contact with other-race individuals would be more inclined to focus on the nose of the own-race Chinese faces than that of the other-race Caucasian faces, similar to those infants in Liu et al. [23].

## Methods

### Participants

The participants were 40 right-handed undergraduate students (23 males) aged from 20 to 25 years (Mean age  = 23.2 years, *SD* = 1.49 years). All were native Chinese without any direct contact with Caucasian or other non-Chinese individuals. The study was conducted according to the NIH research ethical guidelines and approved by Zhejiang Normal University Research Ethics Review Committee. Participants gave written informed consent prior to their participation and were compensated for their participation.

### Materials

**Figure 1:**
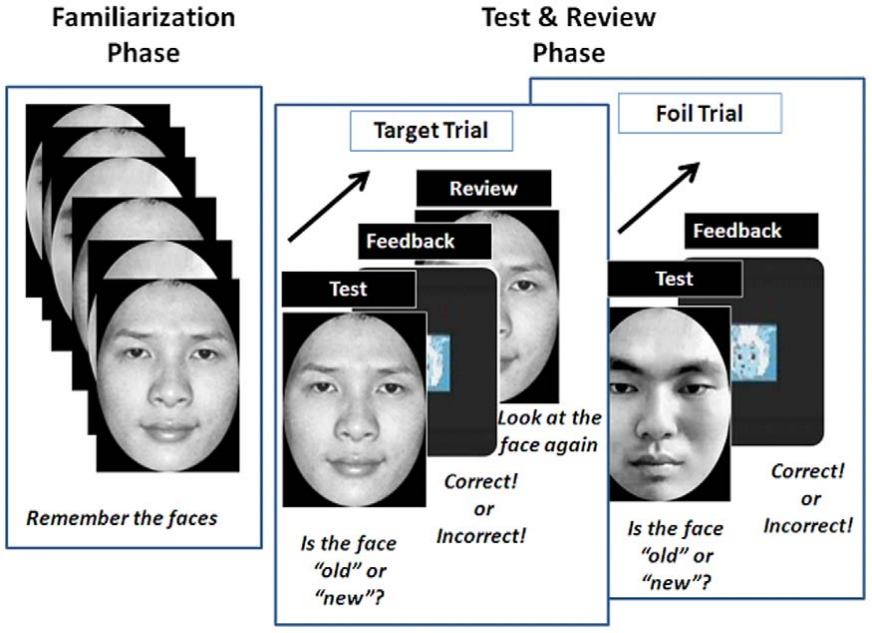
Schematic representation of the experimental design.

Forty photos of Caucasian faces (20 male) and forty photos of Chinese faces (20 male) were used (width: 500 pixels, 13.5 centimeters, 12.7 degrees of visual angle, height: 700 pixels, 18.9 centimeters, 17.9 degrees of visual angle, resolution: 72 pixels per inch). They were all normalized to be the same shape and size. Also, their eyes, nose, and mouth positions were normalized to the locations of the eyes, nose, and mouth of an average face such that the major face features of all face stimuli were located in the same face regions. All face images were frontal view and rendered grey to prevent any differences in skin tone between the Chinese and Caucasian faces from affecting participants' scanning of the faces. To further control for hairstyle differences, all face images were overlaid with the same elliptical shape (Figure 1). The images were matched in overall brightness and luminance using Photoshop. Further, the faces were chosen according to the results of a matching experiment such that the Caucasian and Chinese faces were matched in terms of attractiveness and distinctiveness as judged by Chinese and Caucasian adults who did not participate in the current study. We only selected faces that were judged similarly by Caucasian and Chinese adults in terms of distinctiveness and attractiveness. This selection criterion controlled for potential confounds of facial distinctiveness and attractiveness on participants' recognition performance and scanning patterns. However, one drawback of this stimulus selection method was that it might potentially reduce or eliminate the other-race recognition effect.

A Tobii 1750 Eye tracker (0.5 degree precision, 17 inch, 50 Hz sample rate, 5 fps per second, 1280×1024 pixels resolution) was used to record participants' fixations on the face images. The Tobii Studio program was used to control the stimulus presentation.

### Procedure

The participants took part in the study individually. They were positioned 60 cm from the eye-tracker screen. Participants used a mouse connected to the computer running the Tobii Studio program to respond. Response time and accuracy rates were calculated by an in-house program. Fixation data were recorded by the Tobii eye-tracker automatically. Participants took part first in a practice phase. Four photos (2 Caucasian and 2 Chinese, one gender for each race) were presented to the participant to be remembered. Then they were mixed with four new faces (again 2 Caucasian and 2 Chinese, one gender for each race). The participants judged whether they were old or new faces. All participants understood the experimental task as evidenced by their perfect scores during the practice phase. The faces shown in the practice phase were never shown again in the experimental phase.

Before the experimental phase, the eye movements of participants were calibrated. The calibration program asked participants to follow a bouncing red dot with their eyes as it moved around the screen. The diameter of the red dot was changing from 0 to 1 inch. If the participant's fixation was more than 1 inch away from the center of the dot, a re-calibration was performed. Once the calibration was successful, the familiarization block (Block 0) of the experimental phase began. The results of this calibration were used to calculate the fixation points of the participants in the familiarization block.

The experimental phase consisted of one familiarization block (Block 0) and five test blocks (Blocks 1–5). In the familiarization block, participants were shown the 12 target faces (6 Chinese and 6 Caucasian, 3 males and 3 females for each race). The faces were randomly chosen from the set of 80 faces for each participant. They were shown for 3 seconds each followed by a cartoon character used as a mask (2 seconds). The cartoon character also announced “the next image”.

After all 12 images were presented, the experimenter initiated the first test block. At the beginning of this test block, the above calibration procedure was run again and the result of this calibration was used to calculate the fixation points of the participants in the first test block. Then, the familiarized target face (old face) or a new foil face was shown. Participants were instructed to respond as fast and as accurately as possible by pressing a key to indicate whether it was an old or new face (Test). As soon as participants responded, the cartoon character appeared for 2 seconds, announcing whether the face was indeed an old or new face to give feedback to participants (Feedback). If the preceding face was a target face, the cartoon face reappeared and announced that the face would be shown again, after which the target face just seen would be shown for 30 seconds for participants to review (Review). The next test trial began. If the preceding face was a new foil face, the cartoon character appeared for 2 seconds, announcing whether the face was indeed an old or new face (Feedback), but the foil face in the foil trial was not reviewed. Immediately, the cartoon character announced “the next image”, after which a new trial began. This test-feedback-review (test-feedback) cycle would be repeated until all 12 target faces and 12 foil faces were shown (24 trials in total). The order of the target and foil faces was randomized.

Once the first block was completed, the participants were given a break for about 1 minute to avoid fatigue. The next block then began, also with the calibration procedure first followed by 24 trials. The calibration results of each block were used for calculating the fixation points of the participants in each of the blocks. In total, 5 blocks were run. For each block, the target faces were the same but the foil faces were different and never repeated. Also, the blocks in which the foil faces were presented were counter-balanced between subjects such that in each block they were different for different participants.

## Results

### Discriminating ability, normalized criterion c, accuracy, and correct response time

**Figure 2:**
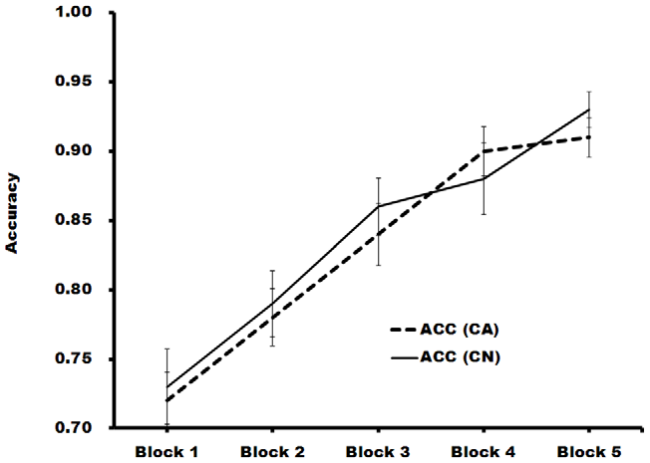
Accuracies of own- and other- race face recognition as a function of trial blocks.

The means and standard deviations for participants' accuracy (%), discriminating ability d', normalized criterion c, and correct response time (ms) for Chinese and Caucasian faces in each test block are presented in Table 1. A 2 (face race) × 5 (test block) repeated measures ANOVA was run on accuracy. Only test block effect was significant, *F*(4,156)  = 47.69, *p<*0.001, *η* ^2^  = 0.55: with increased test blocks, participants became increasingly more accurate in differentiating the old and new faces. Note that the adjusted *F* value, *p* value and degrees of freedom were used henceforth when the Mauchly's Test of Sphericity was significant. A 2 (face race) × 5 (test block) repeated measures ANOVA was run on participants' discriminating ability d's. Only test block effect was significant, *F*(4,156)  = 47.28, *p<*0.001: with increased test blocks, participants became increasingly capable of differentiating the old and new faces (Figure 2.)

**Table 1:** Discriminability, normalized criterion, accuracy, and correct response time.

Test Block	ACC (CN)	ACC (CA)	d' (CN)	d' (CA)	C (CN)	C (CA)	CRT (CN)	CRT (CA)
**1**	0.73 (0.17)	0.72 (0.13)	1.55 (1.38)	1.48 (1.02)	0.16 (0.53)	0.16 (0.5)	1594.65 (583.87)	1658.96 (580.43)
**2**	0.79 (0.15)	0.78 (0.13)	2.12 (1.19)	1.92 (1.02)	0.04 (0.44)	0.08 (0.47)	1532.03 (528.2)	1581.06 (559.44)
**3**	0.86 (0.13)	0.84 (0.14)	2.71 (1.15)	2.51 (1.18)	−0.05 (0.39)	0.05 (0.31)	1429.6 (516.33)	1486.18 (516.43)
**4**	0.88 (0.16)	0.9 (0.11)	2.92 (1.34)	2.96 (1.11)	−0.1 (0.27)	0.03 (0.19)	1339.51 (443.79)	1389.03 (497.87)
**5**	0.93 (0.08)	0.91 (0.09)	3.3 (0.85)	3.16 (0.9)	−0.02 (0.15)	0.01 (0.21)	1276.94 (440.96)	1302.43 (333.39)

(Note: CN  =  Chinese faces, CA  =  Caucasian faces, d'  =  discriminating ability, c  =  normalized criterion c, ACC  =  accuracy, CRT  =  correct response time in miliseconds)

A 2 (face race) × 5 (test block) repeated measures ANOVA was also run on normalized criterion c. Again, only test block effect was significant, *F*(4, 128)  = 5.33, *p = *0.003, *η* ^2^ = 0.14: Initially, the participants were slightly biased towards saying “old” but with increased test blocks, participants' responses become increasingly unbiased. Finally, a 2 (face race) × 5 (test block) repeated measures ANOVA was run on the correct response time. Test block effect was significant, *F*(4, 156)  = 18.68, *p<*0.001, *η* ^2^ = 0.32: with increased blocks, participants became increasingly faster in their correct responses.

Pearson correlations were computed between these measures and the eye tracking measures (below) in each block and none was significant.

### Total fixation time on the target and foil faces

Recall that the participants saw the target Chinese and Caucasian faces initially once during the familiarization phase (Block 0), again during the test period of the recognition phase, and then once more during the review period of the recognition phase of each block (Blocks 1 to 5).

Participants spent most of the time on the screen: The mean rate of fixations on the screen when Chinese faces were shown was 94.4% (SD = 3.0%) and 94.1% (SD = 3.5%) when Caucasian faces were shown. The difference between the two types of faces was not significant. Also, because we defined a fixation as any sustained fixation in an AOI for more than 100 ms, any fixations shorter than this criterion would have not been counted as a fixation. However, the rates for such short fixations were small: the mean rate of fixations with duration shorter than 100 ms when Chinese faces were shown was 0.8% (SD = 0.2%), and 0.7% (SD = 0.2%) when Caucasian faces were shown. The difference between the two types of faces was not significant.

Table 2 and Table 3 shows the means and standard deviations of the total valid fixation time on the face area of the screen in these periods. The total duration of valid fixations on the Chinese faces (M = 179.35 s, SD = 30.76 s) or the Caucasian faces (M = 181.99 s, SD = 27.37 s) were not significantly different.

**Table 2:** The mean and standard deviations of the total fixation time (standard deviation) on the target Chinese and Caucasian faces.

Phase	Face Stimulus	
	Chinese Face	Caucasian Face
Familiarization Phase (Block 0)	2984.2(35.04)	2910.88(31.7)
Review period of Block 1	2870.68(65.96)	2898.56(52.1)
Review period of Block 2	2873.28(58.42)	2879.93(61.27)
Review period of Block 3	2865.07(48.68)	2848.67(54.62)
Review period of Block 4	2826.43(63.72)	2752.55(59.41)
Review period of Block 5	2783.88(72.41)	2812.25(52.56)
Recognition period of Block 1	1895.13(135.69)	1878.23(120.01)
Recognition period of Block 2	1675.36(90)	1711.09(104.31)
Recognition period of Block 3	1584.95(90.38)	1642.05(104.77)
Recognition period of Block 4	1432.16(92.46)	1518.62(90.21)
Recognition period of Block 5	1353.2(86.98)	1510.78(71.83)

**Table 3:** The mean and standard deviations of the total fixation time (standard deviation) on the foil Chinese and Caucasian faces.

Phase	Face Stimulus	
	Chinese Face	Caucasian Face
Recognition period of Block 1	1757.73(105.37)	1824.82(105.64)
Recognition period of Block 2	1806.26(111.49)	1813.08(103.96)
Recognition period of Block 3	1648.57(101.31)	1704.29(113.11)
Recognition period of Block 4	1460.41(79.36)	1569.05(77.78)
Recognition period of Block 5	1508.8(90.88)	1463.89(69.14)

#### Target face in the familiarization phase and review periods of the recognition phase

Because the familiarization phase and the review period of the recognition phase were of the same length (3 seconds), we conducted a 2 (face race) × 6 (test block: 0 to 5) ANOVA on the data of these two periods. There was no significant effect or interaction. Thus, the participants spent the same amount of time on the same target Chinese and Caucasian faces and their attention to the faces did not change with advancing test blocks.

#### Target face in the test periods of the recognition phase

During the test period of the recognition phase of each block, the viewing time was controlled by the participants, and was terminated as soon as the participants responded “old” or “new”. A 2 (face race) × 5 (test block) was conducted on the data. Only the test block effect was significant, *F*(4, 156)  = 11.38, *p<*0.001, *η* ^2^ = 0.23: with increased test blocks, participants' scanning time of the face before their decision decreased significantly. It suggested that as the experiment progressed, participants took less time to report the recognition of the familiarized faces. This was expected because they were given feedback about their performance and re-familiarized with the target faces in each block.

#### Foil face in the test periods of the recognition phase

Table 3 shows the means and standard deviations of the total fixation time on the foil Chinese and Caucasian faces in the test period of the 5 test blocks. A 2 (face race) × 5 (test block) was conducted on the data. Only the test block effect was significant, *F*(4, 156)  = 8.56, *p<*0.001, *η* ^2^ = 0.18: with increased age, participants' scanning time of the face before decision decreased.

### Fixation proportion on the eyes, nose, and mouth

In order to examine more appropriately participants' fixations on the AOIs (eyes, nose, mouth), we used a proportion fixation time measure. This measure was obtained by dividing the sum of the fixation time on each of the AOIs by the total fixation time on the whole face. The mean total fixation proportion on all the AOIs (eyes, nose, mouth) combined was 63.0% (*SD* = 12.7%) for Chinese faces, and 64.5% (*SD* = 12.6%) for Caucasian faces. The difference was not significant, *t = *1.785, *df* = 39, *p = *0.082. It should be noted that the total fixation time on all AOIs (eyes, nose, mouth) did not add to 100% of the on-face fixation duration because when participants fixated on the face area of the stimulus, their fixations might still fall outside of the four AOIs.

**Figure 3:**
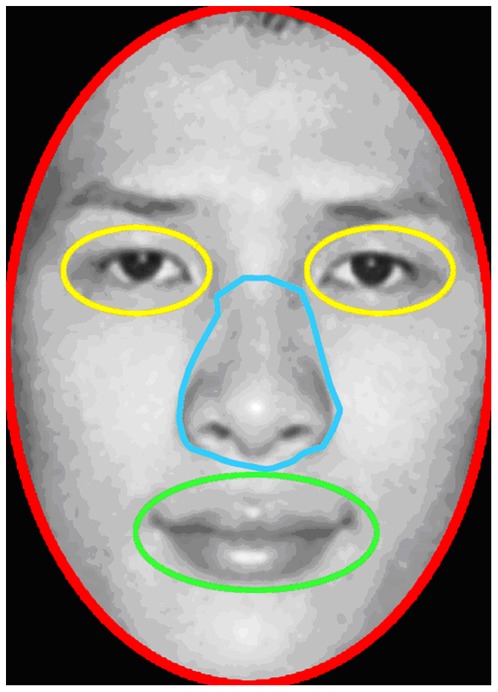
Example of areas of interest (AOI) plots.

Because participants scanned different faces with different amounts of total fixation time, in order to examine more equitably whether participants fixated on the key features of the Chinese and Caucasian faces differently (i.e., eyes, nose, and mouth), we first defined a number of areas of interest (AOIs) for each face of each race: the whole face (the area within the face contour), the eyes (right and left combined), the nose, and the mouth (for an example, see Figure 3). Second, we obtained the total fixation time on each of the AOIs. Third, we computed the proportional fixation time on the AOIs of the eyes, nose, and mouth for each face of each race by dividing the total fixation time on the eyes, nose, or mouth of a particular face by the total fixation time on the face. Fourth, because the physical sizes of the three AOIs differed slightly, we adjusted the above-mentioned proportional fixation times on the eyes, nose, and mouth of each face to account for the AOI size differences. This was done by having the size of the eye AOI, nose AOI, and mouth AOI divided by the size of the mouth AOI, which in effect used the mouth AOI size as a reference. This adjustment was done for each face. It should be noted that the three AOIs were generally similar in size and thus the proportional fixation time on the three AOIs before and after the adjustments were highly similar. Nevertheless, we used the adjusted proportional fixation time data for the subsequent analyses (henceforth referred to as fixation proportion).

**Figure 4:**
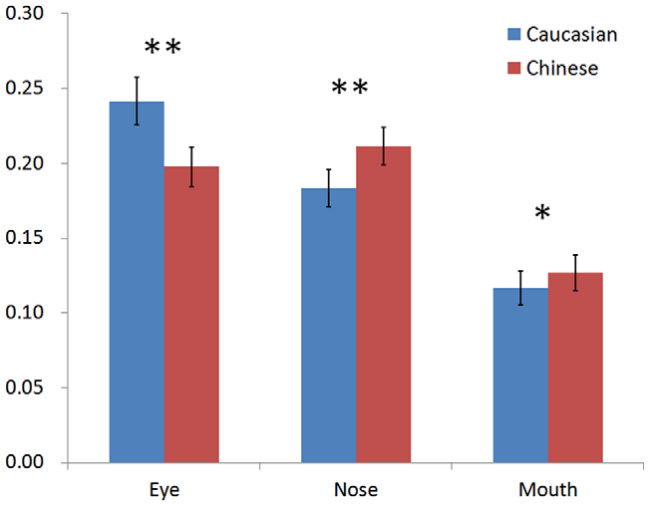
Mean fixation proportion on the eyes, nose and mouth during the familiarization and review of the target faces. (Note: *p<0.05; **p<0.01).

Table 4 shows the means and standard deviations of the fixation proportion on the eyes, nose, and mouth of the Chinese and Caucasian target faces in the familiarization phase (Block 0) and the review periods of the recognition phase (Blocks 1 to 5). A 2 (face race) × 3 (face region) × 6 (viewing period) ANOVA was performed on the fixation proportion density. The effects of face region and block were significant, *F*(2, 78)  = 13.41, *p<*0.001, *η* ^2^ = 0.26 and *F*(5, 195)  = 3.17, *p = *0.024, *η* ^2^ = 0.08, respectively. The crucial interaction between face race and region was significant, *F*(2, 78)  = 39.04, *p<*0.001, *η* ^2^ = 0.5. Post hoc pair wise t-tests revealed that participants spent significantly more time on the eyes of the Caucasian faces than the Chinese faces, *t = *−6.73, *df = *39, *p<*0.001 (Figure 4). In contrast, they spent significantly more time on the nose of the Chinese faces than the Caucasian faces, *t = *5.01, *df* = 39, *p<*0.001 (Figure 4). As for the mouth, they spent significantly more time on Chinese mouth, *t = *2.26, *df = *39, *p = *0.029 (Figure 4).

**Table 4:** Mean fixation proportions for different ROIs of Chinese and Caucasian target faces in the Familiarization Phase (Blocks 1–5).

	Eyes			Nose			Mouth		
Block	CN	CA	t	CN	CA	t	CN	CA	t
0	0.20 (0.11)	0.24 (0.12)	−3.23**	0.22 (0.10)	0.19 (0.11)	2.90**	0.14 (0.10)	0.14 (0.10)	0.41
1	0.19 (0.10)	0.26 (0.13)	−4.90**	0.22 (0.11)	0.182 (0.10)	3.09**	0.13 (0.08)	0.11 (0.08)	1.52
2	0.19 (0.09)	0.25 (0.12)	−4.80**	0.193 (0.09)	0.18 (0.08)	2.07**	0.14 (0.09)	0.11 (0.08)	3.02**
3	0.21 (0.10)	0.24 (0.12)	−2.54*	0.214 (0.11)	0.177 (0.09)	2.34**	0.13 (0.11)	0.13 (0.09)	0.18
4	0.21 (0.13)	0.24 (0.13)	−1.89	0.19 (0.10)	0.19 (0.11)	0.16	0.11 (0.09)	0.10 (0.07)	0.45
5	0.17 (0.09)	0.21 (0.12)	−2.50*	0.23 (0.10)	0.19 (0.10)	3.43**	0.11 (0.10)	0.11 (0.10)	0.17
**Total**	0.19 (0.08)	0.24 (0.10)	−6.73**	0.21 (0.08)	0.18 (0.08)	5.01**	0.13 (0.07)	0.12 (0.07)	2.26*

(Notes: *p<0.05, **p<0.01, CN  =  Chinese faces, CA  =  Caucasian faces, Total  =  all blocks combined).

**Figure 5:**
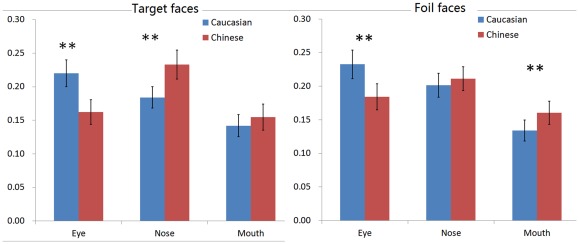
Mean fixation proportion on the eyes, nose and mouth during the recognition of the target and foil faces. (Note: **p<0.01).

Table 5 shows the means and standard deviations of the fixation proportion on the eyes, nose, and mouth of the Chinese target and Caucasian target faces during the test periods of the recognition phase (Blocks 1 to 5). A 2 (face race) × 3 (face region) × 5 (test block) ANOVA was performed on the data. Only the interaction between face race and face region was significant, *F*(2, 78)  = 26.98, *p<*0.001, *η* ^2^ = 0.41. Post hoc pairwise t-tests revealed that participants spent significantly more time on the eyes of the Caucasian faces than the Chinese faces, *t = *−6.15, *df = *39, *p<*0.001 (Figure 5). In contrast, they spent significantly more time on the nose of the Chinese faces than the Caucasian faces, *t = *4.45, *df = *39, *p<*0.001 (Figure 5). As for the mouth, there was no significant difference, *t = *1.72, *df = *39, *p = *0.094 (Figure 5).

**Table 5:** Mean fixation proportions for different ROIs of Chinese and Caucasian target faces in the Test Periods of the Recognizing target faces Phase (Blocks 1–5).

	Eyes			Nose			Mouth		
Block	CN	CA	*t*	CN	CA	*t*	CN	CA	*t*
**1**	0.18 (0.14)	0.22 (0.15)	−2.76**	0.21 (0.14)	0.20 (0.13)	0.87	0.17 (0.13)	0.17 (0.12)	0.53
**2**	0.16 (0.13)	0.22 (0.15)	−4.71**	0.21 (0.15)	0.18 (0.12)	1.81	0.16 (0.16)	0.15 (0.14)	1.12
**3**	0.16 (0.11)	0.21 (0.14)	−3.16**	0.24 (0.17)	0.17 (0.11)	3.65**	0.15 (0.14)	0.16 (0.14)	−0.76
**4**	0.16 (0.14)	0.23 (0.16)	−5.18**	0.25 (0.18)	0.19 (0.13)	3.34**	0.16 (0.16)	0.14 (0.19)	1.55
**5**	0.16 (0.15)	0.23 (0.16)	−3.08**	0.25 (0.15)	0.18 (0.19)	3.59**	0.13 (0.13)	0.10 (0.11)	1.93
***Total***	0.16 (0.12)	0.22 (0.13)	−6.15**	0.23 (0.14)	0.18 (0.10)	4.45**	0.15 (0.12)	0.14 (0.10)	1.72

(Notes: **p<0.01, CN  =  Chinese faces, CA  =  Caucasian faces, Total  =  all blocks combined).

Table 6 shows the means and standard deviations of the fixation proportion on the eyes, nose, and mouth of the Chinese foil and Caucasian foil faces during the test periods of the recognition phase (Blocks 1 to 5). We performed a 2 (face race) × 3 (face region) × 5 (test block) ANOVA. Test block effect was significant, *F*(4, 156)  = 2.46, *p = *0.048, *η* ^2^ = 0.06. The crucial interaction between face race and face region was significant, *F*(2, 78)  = 30.37, *p<*0.001, *η* ^2^ = 0.44. Post hoc pairwise t-tests revealed that participants spent significantly more time on the eyes of the Caucasian faces than the Chinese faces, *t = *−6.36, df = 39, *p<*0.001 (Figure 5B). In contrast, they spent significantly more time on the mouth of the Chinese faces than the Caucasian faces, *t = *4.54, *df = *39, *p<*0.001 (Figure 5). As for the nose, there was no significant difference, *t = *1.63, *df = *39, *p = *0.112 (Figure 5).

**Table 6:** Mean fixation proportions for different ROIs of Chinese and Caucasian foil faces in the Test Periods of the Recognizing Foil faces Phase (Blocks 1–5).

	Eyes			Nose			Mouth		
Block	CN	CA	*t*	CN	CA	*t*	CN	CA	*t*
**1**	0.18 (0.14)	0.23 (0.15)	−4.25**	0.22 (0.12)	0.21 (0.12)	0.5	0.17 (0.11)	0.15 (0.13)	0.87
**2**	0.19 (0.13)	0.23 (0.15)	−3.23**	0.18 (0.11)	0.20 (0.16)	−1.22	0.18 (0.15)	0.13 (0.11)	4.04**
**3**	0.19 (0.15)	0.25 (0.15)	−2.73**	0.21 (0.14)	0.19 (0.15)	1.29	0.17 (0.15)	0.15 (0.14)	1.3
**4**	0.19 (0.15)	0.236 (0.16)	−3.22**	0.22 (0.14)	0.19 (0.11)	1.8	0.16 (0.13)	0.14 (0.12)	1.11
**5**	0.17 (0.15)	0.23 (0.18)	−3.01**	0.22 (0.15)	0.21 (0.16)	0.85	0.13 (0.12)	0.11 (0.11)	1.57
***Total***	0.18 (0.12)	0.23 (0.13)	−6.36**	0.21 (0.11)	0.20 (0.11)	1.63	0.16 (0.11)	0.13 (0.10)	4.54**

(Notes: **p<0.01, CN  =  Chinese faces, CA  =  Caucasian faces, Total  =  all blocks combined).

### Raw fixation difference map

To further explore the fixation data on own- and other-race faces, we used the iMap Matlab toolbox, a novel method that computes statistical fixation maps of eye movements [26]. Unlike the above AOI analyses that amalgamate all fixation points that fall into a particular predetermined area of interest and then perform statistical tests on the total fixations to the area between conditions, iMap allows for statistical testing of condition differences on any part of a stimulus without the restriction of the AOIs. Also, it allows for statistical testing of condition differences on a scale finer than the AOI analyses.

**Figure 6:**
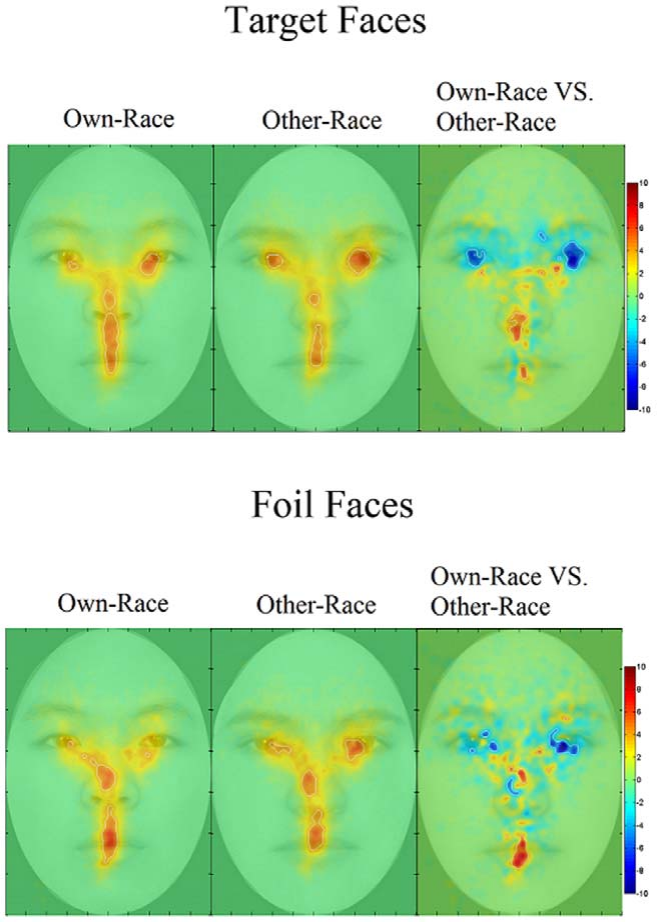
Participants' Raw fixation maps in Z values for viewing own-race Chinese target faces (first column), other-race Caucasian target faces (second column), and the difference between viewing own-race and other-race faces (third column). Areas showing significant fixations are delimited by white borders. (p<0.05, corrected).

All participants' fixation data on all Chinese and Caucasian target faces were analyzed with the iMap method, from which we obtained raw fixation maps (Figure 6) in T value for Chinese faces (first column) and for Caucasian faces (second column), and raw fixation difference map in T value for Chinese faces – Caucasian faces (third column). Areas showing a significant fixation difference are delimited by white borders (p<.05, corrected). In the third column of Figure 6, hot colors (i.e., red) denote greater fixations on Chinese faces than Caucasian faces and cold colors (i.e., blue) denote greater fixations on Caucasian faces than Chinese faces. Values near 0 (or white color) indicate similar magnitude in fixation between the faces of the two races.

Consistent with the AOI analysis findings, the iMap analysis shows that Chinese participants fixated more on the eye regions of the Caucasian faces. In particular, they appeared to fixate on the pupils of the Caucasian eyes significantly more than those of the Chinese eyes. In contrast, the participants fixated significantly more on the midline of the Chinese faces than that of the Caucasian faces, starting just below the nasal bridge. In particular, they fixated on the top and base (columella) of the nose, the philtrum (i.e., the area between the nose and mouth), and the center of the lower lip of the Chinese faces significantly more than those of the Caucasian faces.

It should be noted, however, that as shown in the raw fixation difference map, although Chinese participants fixated significantly less on the eyes of the own-race faces, they did fixate significantly more on the regions just below the eyes, as if to avoid direct eye contact. Also, analyses of the fixation distributions showed that fixations moved downward rather than being more concentrated. Also, although the participants generally fixated on the nose of Chinese faces more than that of Caucasian faces, significantly greater fixation on the Caucasian nose than the Chinese nose was still found on the tip of the nose. However, the greater fixations on the Chinese nose appeared to be more widespread than those on the Caucasian nose.

### Stimulus salience analysis

**Figure 7:**
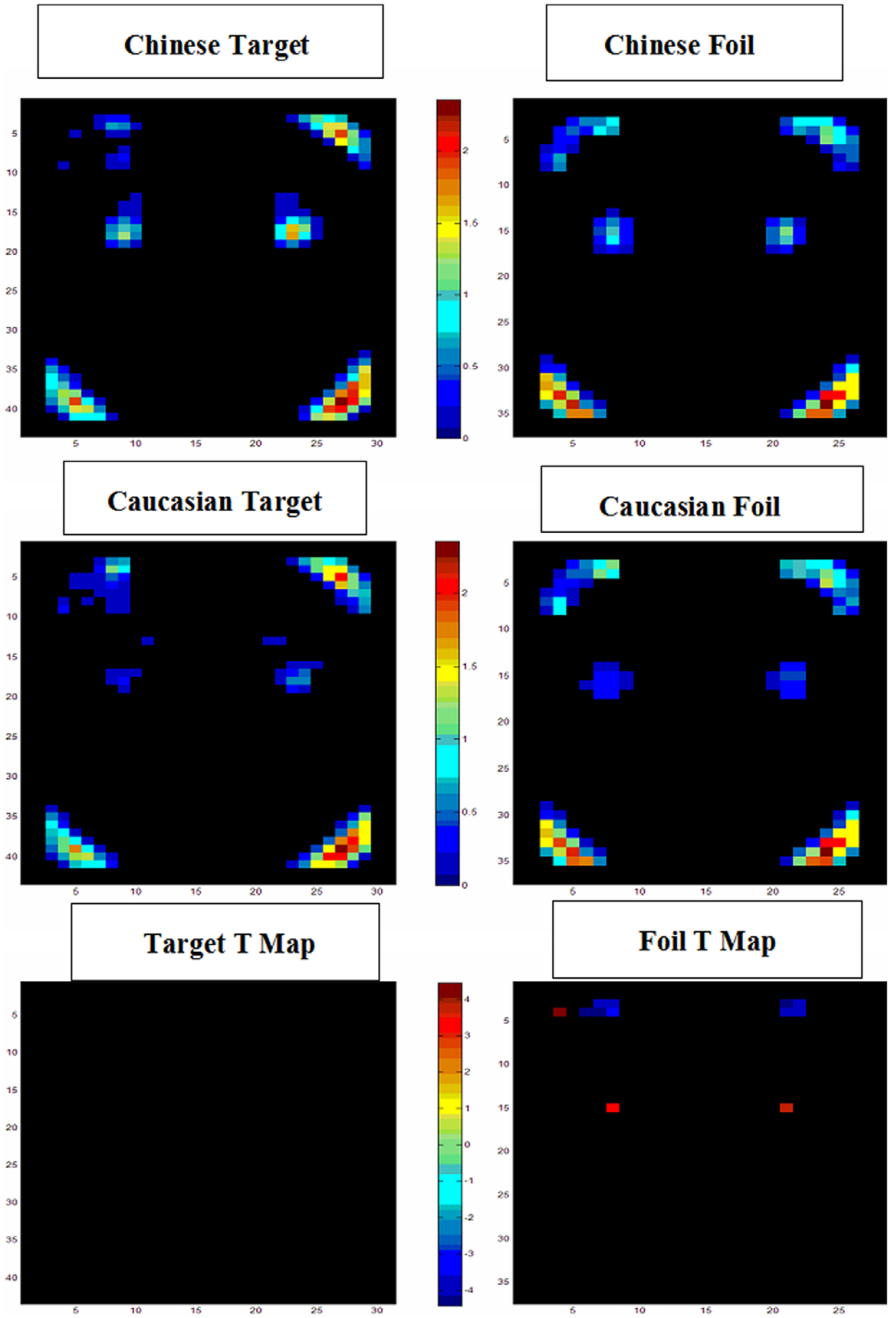
Mean saliency maps for the Chinese target and foil faces, Caucasian target and foil faces, and the significant difference T-maps (Chinese face saliency – Caucasian face saliency). X and Y axes represent the mean horizontal and vertical coordinates of each pixel of the Chinese or Caucasian faces (as measured in the proportion of the corresponding axis of a face). The colors on the top two temperature bars refers to the mean saliency values of the Chinese or Caucasian faces, with warm colors denoting high saliency and cold colors denoting low saliency. The colors on the bottom temperature bar refer to the T value of the difference in saliency between the Chinese and Caucasian images, with warm color denoting positive T values (Chinese faces more salient than Caucasian faces) and cold colors denoting negative T values (Caucasian faces more salient than Chinese faces). Only significant T values are shown (FDR corrected).

One possibility for participants' differential attention to the Chinese and Caucasian eyes, noses, and mouth might be that the Chinese and Caucasian faces have different perceptual salience such that participants' visual attention was naturally drawn to the salient eyes in the Caucasian faces and the salient nose and mouth in the Chinese faces. To test this possibility, we performed a saliency analysis using the Saliency Toolbox designed by Walther and Koch [27]. This toolbox can calculate saliency for each area in a photo based on a psychologically plausible neural network model. This model is built on the assumption that more directed selective attention should be paid to areas with greater salience for better recognition [25]. During the process, each face photo was automatically divided into 37*28 grids. The saliency results of each of the Chinese face photos were spatially averaged to derive a mean saliency map for the Chinese target and foil faces, and so were those of the Caucasian target and foil faces (Figure 7). Then, the grids in the Chinese and Caucasian face regions were compared using the “gene mattest” procedure (independent t-tests) in Matlab2010a, variance assumed to be unequal. Grids on the borders of the faces were not counted in the analysis.

Regarding the target faces, the saliency analyses revealed that the target Chinese and Caucasian faces are highly similar in their salience patterns. They are highly salient in the eye regions. Interestingly, the nose and mouth regions for both Chinese and Caucasian faces are not salient. Regarding the salience differences between the target Chinese and Caucasian faces, after adjustments for type I error using the FDR method, there were no significant differences in saliency between the Chinese and Caucasian target faces in any part of the face images (Figure 7).

For the foil faces, the saliency analyses revealed patterns of saliency similar to those of the target faces: the foil Chinese and Caucasian faces are highly salient in the eye regions and the four corners of the face contour. Again, the Chinese and Caucasian foil faces are not salient in their nose and mouth regions. When the salience maps of the Chinese foil faces were contrasted with those of the Caucasian foil faces, the Caucasian faces were significantly more salient than the Chinese faces in the upper face contour regions, whereas the Chinese foil faces were more salient only in 2 grids (one in the right eye region and the other in the left eye region) than the corresponding regions of the Caucasian foil faces, all *t*>32.45, *df* = 58, all *q*<0.005, all *p*<0.002 (Figure 7).

It should be noted that the four corners of the face contour were highly salient too. They were in fact the edges where the face overlaps with the oval. Because we used the same ellipse to encircle all faces, there was no variability in information outside the ellipse. The saliency results thus merely reflected contrasts between the edges of the encircled faces. However, this change did not affect the saliency results of the eyes, nose, and mouth. Thus, the saliency results around the border of the ellipse did not meaningfully reflect the saliency of the face stimuli themselves and should be ignored (Ideally, we should only submit the encircled faces to the analyses without the border regions, but due to a limitation of the toolbox, a square must be submitted).

## Discussion

The present study tested the hypothesis that individuals would scan own- and other-race faces differently. Results strongly supported this hypothesis. When given a fixed amount of time to view target Chinese or Caucasian faces for familiarization or for review, Chinese participants spent a significantly greater proportion of fixation time on the eyes of other-race Caucasian faces than the eyes of own-race Chinese faces. In contrast, they spent a significantly greater proportion of fixation time on the nose and mouth of Chinese faces than the nose of Caucasian faces (Figure 4). As shown in Table 4, this pattern of differential fixation, for own- and other-race eyes and mouth in particular, was consistent even as participants became increasingly familiar with the target faces. Additionally, the raw fixation difference map provided by the iMap Matlab toolbox confirmed these findings.

To provide further evidence for the robustness of the differential scanning patterns for own-race Chinese faces and other-race Caucasian faces, we found that when participants were allowed to terminate their scanning at any point during the recognition of the familiarized target faces, Chinese participants again proportionally fixated on the eyes of Caucasian faces significantly more than those of Chinese faces, whereas they fixated on the nose of Chinese faces significantly more than the nose of Caucasian faces. As shown in Table 5, with increased familiarity with the target faces, the focus on the eyes of Caucasian faces remained significantly greater than on the eyes of Chinese faces. In contrast, although there were no significant differences in the proportion of fixation time on the nose of own- and other-race noses in the initial two blocks, participants fixated on the Chinese nose significantly more than the Caucasian nose after they became familiarized with both types of target faces.

The above findings regarding the scanning patterns on the target faces could not be explained by potentially different amounts of time that participants might spend on the own- and other-race faces. In fact, participants spent equal amounts of time on the own- and other-race target faces during the familiarization period, the review period, and the testing period. Nor could the findings be explained by the perceptual salience of the Chinese nose or mouth versus that of the Caucasian eyes. When we applied the Saliency Map procedure to the target face stimuli used in the present study, the eye regions of the Caucasian target faces were not any more perceptually salient than those of the Chinese target faces, nor were there any differences between the Chinese and Caucasian target faces in perceptual salience in the nose or mouth regions (Figure 7). Furthermore, the above findings cannot be explained by potential differences in recognition performance of the own- and other-race faces. When assessed in terms of accuracy, discriminability, response biases, and response latency, participants' performance was highly comparable for own- and other-race faces.

In many previous studies, participants tended to recognize faster and better own-race faces than other-race faces [1]–[3]. However, unlike the present study, those studies tended to use more target faces and/or shorter viewing time, typically without feedback, which made the task more challenging than the task used in the present study [7]. We changed these experimental parameters for the present study to ensure that we could collect a sufficient amount of fixation data for analysis. One problem of this methodological change is that our results may not be able to account for the robust other-race effect in recognition performance consistently reported in the literature. Note that in the present study, although Chinese participants used different scanning strategies for own- and other-race faces, their recognition performance for both types of faces was nearly identical. We failed to find any significant correlations between the use of the nose- or eye-centric scanning strategies and the level of recognition performance. It is thus unclear whether the visual scanning strategies would have any direct impact on memory performances of own- and other-race faces. Specifically designed studies are still needed to increase the task difficulty so as to obtain the robust other-race effect in behavior, which would then make it possible to examine the linkages between participants' visual scanning patterns and the other-race effect in recognition. One possible outcome may be that whereas the eye tracking patterns will be different for own- and other-race faces similar to the present findings, the recognition accuracies for own- and other-race faces will be correlated with the fixation measures. Another possibility is that even when the other-race effect is observed behaviorally, the fixation measures will be uncorrelated with the behavioral performance. In this case, one may need to explore other measures of eye tracking such as scanpath to identify the eye tracking contributors to the ORE [28], [20]. Additionally, Caldara and his colleagues [22] suggested that the nose-centric strategy might facilitate holistic processing, whereas the eye-centric strategy might facilitate featural processing. To test this hypothesis, future studies need to modify the current behavioral paradigm such that participants will be required to use either holistic or featural strategies to process own- and other-race faces.

With regard to the foil faces, the results of the fixation proportion on the eyes were highly similar to those for the target faces: Participants consistently focused on the eyes of the Caucasian foil faces more than the Chinese eyes in all testing blocks. However, their fixation proportion on the nose of own- and other-race faces did not differ in all blocks. Also, although they overall fixated significantly more on the mouth of the own-race faces than on the mouth of the other-race faces, this significant effect was only carried by one block. In all other blocks, no cross-race difference was observed. It is unclear as to why the foil faces only replicated the eye effect for Caucasian faces, but not the nose effect for Chinese faces. When we compared the raw fixation time on the target and foil faces, no apparent differences were observed. Also, the saliency maps for own- and other-race foil faces were not markedly different from those for target faces. When the saliency map of the Chinese foil faces was contrasted with that of the Caucasian foil faces, even though participants focused more on the eyes of the Caucasian foil faces than the eyes of the Chinese foil faces, the Chinese faces were actually slightly more salient than the Caucasian faces in 2 pixels in the eye regions. In contrast, the Caucasian foil faces were only significantly more salient than the Chinese faces in the upper face contour regions. Regardless, participants rarely fixated on these regions and displayed no differential patterns of fixation on these regions between own- and other-race foil faces.

One possibility for the lack of the replication for the nose and mouth of Chinese faces was that we used the same target faces throughout all the blocks whereas we used new foil faces for each testing block. Otherwise, participants might confuse the previously seen foil faces as the target faces. Due to the greater variability of the unfamiliar foil faces, the scanning patterns on the foil faces might have been more variable than those on the target faces. This explanation needs to be verified with specifically designed studies in the future. Nevertheless, despite this inconsistency in findings on the nose, the results regarding the eyes of the foil Caucasian faces attested to the robustness of the general phenomenon of the own- and other-race differential face scanning in Chinese participants.

Our findings (those with the target faces in particular) are in accord with the results obtained from Chinese infants by Liu et al. [23] who found that Chinese infants with increased age became more inclined to scan the nose of the own-race faces more than that of the other-race Caucasian faces [24]. Our findings with Chinese adults may represent the end state outcomes of the developmental course for own- and other-race face scanning that begins in early infancy. As has also been suggested by Liu et al. [23] and Wheeler et al. [24], the differential scanning patterns for own- and other-race faces by Chinese participants are consistent with the enculturation hypothesis [22], [24]. The enculturation hypothesis posits generally that individuals from different cultures may have learned to use different visual strategies for scanning faces due to different cultures' norms governing mutual gaze during social interaction. In many Asian societies (East Asian ones in particular), direct and prolonged eye contact is considered impolite and inappropriate in many contexts [29], [30]. Individuals are socialized to avoid sustained eye contact during social interactions with others. Individuals from very early on are socialized to act according to such eye contact norms. Although the eye contact norms are mainly for regulating face-to-face social interaction (i.e., when viewing live and dynamic faces), Chinese adults with decades of experience using such norms may habitually move gaze away from the eyes of own-race Chinese faces and focus on their noses even when they are simply viewing photographs of static faces. The increased fixation on the nose can be an excellent strategy because the nose as the center of the face allows the viewer the ready access to information on the entire face for the so-called face trait information (e.g., facial featural and configural information for facial identity recognition) and the so-called face state information (e.g., facial emotion, eye gaze, speech [16]).

Because the eye contact norms have been learned for interactions with own-race in-group members, one may not be so constrained to adhere to the norms when viewing other-race faces. Thus, when asked to remember and recognize other-race faces, Chinese participants might scan the faces more freely and be driven by the most salient facial features on the face: As revealed by our saliency analysis of the face stimuli used in the present study, the most salient major face features for both Chinese and Caucasian faces are the eyes (see Figure 7). Perhaps for this reason, our participants tended to focus on the eyes of the other-race Caucasian faces more than that of the Chinese faces for remembering and recognition. If this enculturation hypothesis is true, one should observe developmental changes in the scanning of patterns of own- and other-race faces. Kelly and his colleagues [31] recently examined exactly this issue with school aged Caucasian children in the UK and Chinese children in China. Their results indeed hinted at the developmental tuning of culturally different face scanning strategies.

Our findings appear to be inconsistent with those by Caldara and his associates [22] who reported that Asian participants used a nose-centric scanning pattern regardless of whether the faces seen were Asian or Caucasian, whereas their Caucasian participants used the eye-centric strategy for both Caucasian and Asian faces. In contrast, our study revealed that Chinese participants scanned the nose of own-race target faces more than the nose of other-race faces, and scanned the eyes of other-race faces more than the eyes of own-race faces. One major difference between our study and that of Caldara et al [22]. is that our Chinese participants had no direct contact with any foreign individuals whereas their Asian participants, being newly arrived students in a Scottish university, had some direct interactions with Caucasian individuals likely both on campus and off campus. In other words, their participants had more experiences with Caucasian faces. However, it is entirely unclear as to why the increased exposure to Caucasian faces should lead these Asian participants to generalize their culture-specific scanning strategies to scan Caucasian faces, an issue to be investigated with specifically designed studies.

The present findings also point to other additional important future studies. For example, in our study we only used one type of other-race faces. It is unclear whether Chinese participants would focus on the eye regions only for Caucasian faces or all other-race faces. Also, because we only used Chinese participants, it is unclear whether our findings can be replicated with participants from other cultural backgrounds that practice similar norms (e.g., Japanese in Japan or Africans in Africa) or that do not practice the same eye contact norms (e.g., Caucasians and African Americans living in the US). In addition, although our saliency analyses revealed high similarities between the Chinese and Caucasian faces, there might exist other important differences between the faces of Chinese and Caucasian faces that give rise to the differentiated race-specific face scanning. One possibility is that the Chinese and Caucasian faces differ in physiognomy. Indeed, anthropometric studies of facial morphology between Asian and Caucasian adults [32]–[34] reported major cross-race differences in craniofacial characteristics. When compared with Caucasian faces, Chinese faces have a wider distance between the inner corners of the eyes but a smaller eye width, wider nose, and a smaller mouth width. Although our saliency procedure might not be sensitive enough to detect these unique cross-race differences in facial morphology, such differences, if present, might nonetheless have driven our participants to scan the Chinese and Caucasian faces differently. Thus, additional studies with the same design as ours but involving non-Chinese participants (e.g., Caucasians and Africans), faces from multiple races, and even more sensitive analytic tools, would allow us to address these issues, which in turn should further elucidate the nature of the differences in scanning own- and other-race faces and more broadly the other-race effect.
